# The Role of *gsp* Mutations on the Development of Adrenocortical Tumors and Adrenal Hyperplasia

**DOI:** 10.3389/fendo.2016.00104

**Published:** 2016-07-27

**Authors:** Maria Candida Barisson Villares Fragoso, Ingrid Quevedo Wanichi, Isadora Pontes Cavalcante, Beatriz Marinho de Paula Mariani

**Affiliations:** ^1^Unidade de Suprarrenal, Disciplina de Endocrinologia e Metabologia, Laboratorio de Hormonios e Genetica Molecular LIM/42, Hospital das Clinicas, Faculdade de Medicina da Universidade de Sao Paulo, Sao Paulo, Brazil

**Keywords:** *gsp*, *GNAS*, adrenal, tumors, hyperplasia, mutations

## Abstract

Somatic *GNAS* point mutations, commonly known as *gsp* mutations, are involved in the pathogenesis of McCune–Albright syndrome (MAS) and have also been described in autonomous hormone-producing tumors, such as somatotropinoma, corticotrophoma, thyroid cancer, ovarian and testicular Leydig cell tumors, and primary macronodular adrenocortical hyperplasia (PMAH) (1–3). The involvement of *gsp* mutations in adrenal tumors was first described by Lyons et al. Since then, several studies have detected the presence of *gsp* mutations in adrenal tumors, but none of them could explain its presence along or the mechanism that leads to tumor formation and hormone hypersecretion. As a result, the molecular pathogenesis of the majority of sporadic adrenocortical tumors remains unclear (3). PMAH has also been reported with *gsp* somatic mutations in a few cases. Fragoso et al. identified two distinct *gsp* somatic mutations affecting arginine residues on codon 201 of *GNAS* in a few patients with PMAH who lacked any features or manifestations of MAS. Followed by this discovery, other studies have continued looking for *gsp* mutations based on strong prior evidence demonstrating that increased cAMP signaling is sufficient for cell proliferation and cortisol production (2, 4). With consideration for the previously reported findings, we conjecture that although somatic activating mutations in *GNAS* are a rare molecular event, these mutations could probably be sufficient to induce the development of macronodule hyperplasia and variable cortisol secretion. In this manuscript, we revised the presence of *gsp* mutations associated with adrenal cortical tumors and hyperplasia.

## Introduction

**Figure 1:**
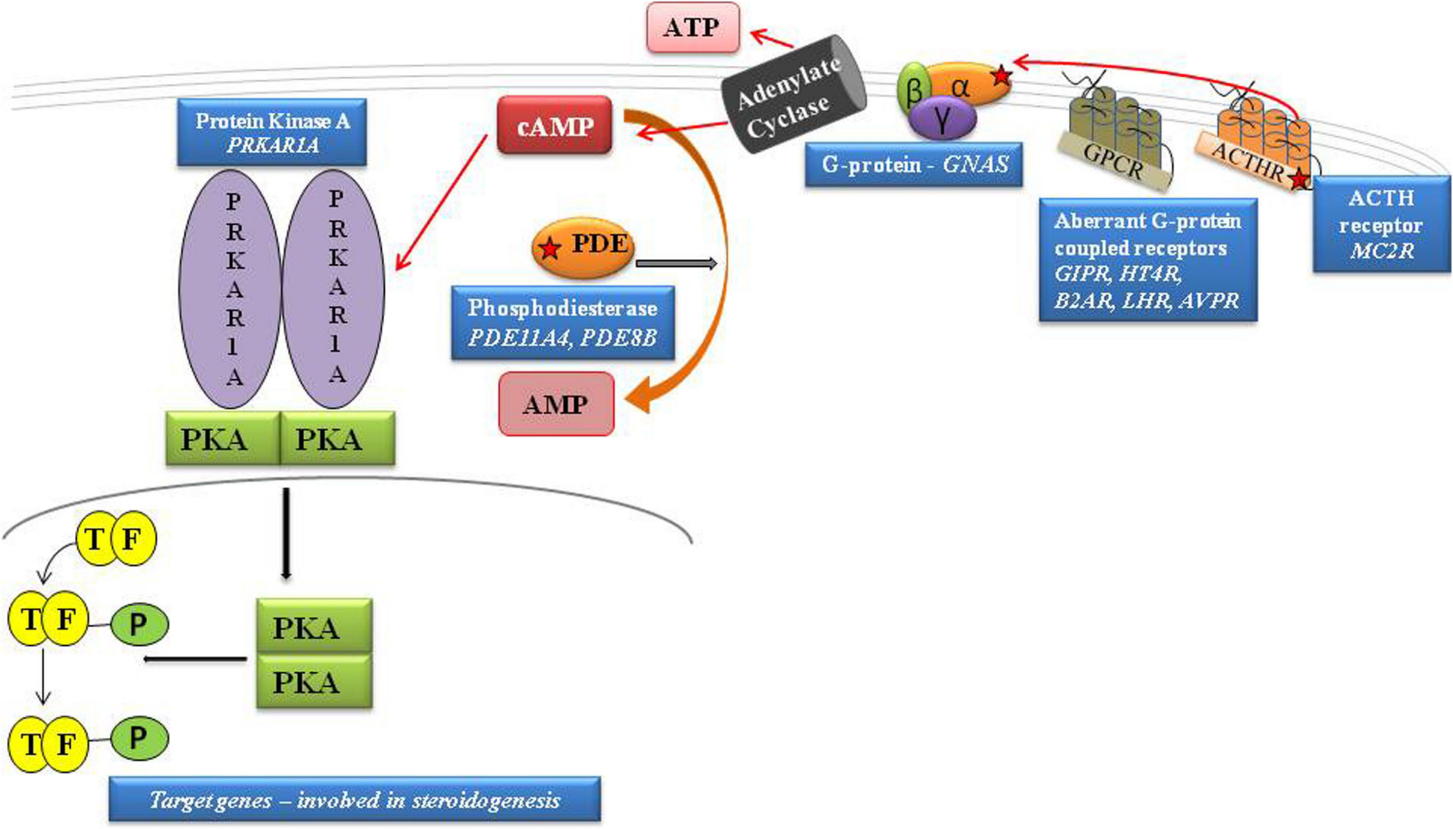
**Schematic representation of cAMP pathway**. GPCRs cause the activation of G proteins by facilitating the exchange of GTP for GDP on the α subunit. cAMP is degraded to AMP by PDEs. PKA is activated by the rise in cAMP that, by binding both high-affinity cAMP-binding sites of each subunit, leads to the release of active subunits that phosphorylate their substrates, including the nuclear transcription factor, CREB. By inhibiting the intrinsic GTPase activity, *gsp* mutations cause constitutive activation of adenylyl cyclase (AC), leading to increased cAMP; protein kinase A activation. The protein kinase A (PKA) induces phosphorylation of the cAMP-binding protein, CREB, facilitating its traslocation to the nucleus and activation of cAMP-responsive gene transcription. [Adapted from Lacroix et al. (11)].

Heterotrimeric G proteins are the molecular switches that turn on intracellular signaling cascades in response to the activation of G protein-coupled receptors (GPCRs) by extracellular stimuli. Therefore, G proteins play a crucial role in defining the specificity and temporal characteristics of the cellular response. Heterotrimeric G proteins consist of three subunits, α, β, and γ, and their switching function depends on the ability of the G protein α-subunit (Gα) to cycle between an inactive GDP-bound conformation that is primed to interact with an activated receptor and an active GTP-bound conformation that can modulate the activity of downstream effector proteins. The α subunit is a GTPase; therefore, when the receptor stimulates the G protein, this subunit releases GDP and binds GTP. In this activated state, several α subunit types act directly on effector molecules to modulate their activity. Some α subunits show specificity for effectors; for example, αs activates adenylate cyclases, αi inhibits adenylate cyclases, and αq activates phospholipase C isozymes (5) (Figure 1).

The gene encoding the alpha subunit of stimulatory G proteins (GNAS, OMIM 139320) is located on chromosome 20q13.32 (6). Activating somatic mutations of stimulatory G protein called *gsp* mutations can result in the loss of intrinsic GTPase activity of the α subunit with subsequent constitutive activation of adenylate cyclase (7, 8).

*gsp* mutations are involved in the pathogenesis of McCune–Albright syndrome (MAS), which is an endocrine disorder that is classically defined by the clinical triad of bone fibrous dysplasia, *café-au-lait* skin, and peripheral precocious puberty (1).

Patients with MAS present with a postzygotic *gsp* mutation in a mosaic distribution, resulting in varying degrees of tissue involvement that range from a single site to a widespread distribution. However, if these mutations were germline, they would be lethal. To date, this concept is supported by the absence of any cases resulting from vertical transmission and the discordance in disease among monozygotic twins (9). Adrenal hypercortisolism affects a minority of patients with MAS due to adrenal nodular hyperplasia and around 20 cases were described (8).

Naturally occurring mutations in codons 201 and 227, which alter the GTPase activity in the *GNAS* gene, have been described in autonomous hormone-producing tumors. Mutations involving substitution of either cysteine or histidine and, more rarely, serine for arginine at codon 201 or arginine for glutamine at codon 227 were first described in GH-producing pituitary tumors (10).

The *gsp* mutations have also been described in several tumors, such as somatotropinoma, thyroid tumor, ovarian and testicular Leydig cell tumors, and primary macronodular adrenocortical hyperplasia (PMAH), as well as in rare cases of corticotropinoma, cortisol, and aldosterone-secreting adrenocortical adenoma. All cases described were outside of the classical presentation of MAS (1, 2, 4, 7).

### cAMP/PKA Signaling in Adrenocortical Cells

The discovery of the role of cAMP (adenosine 3′5′-cyclic monophosphate) as an intracellular mediator introduced the concept of second messengers in signal transduction. cAMP is a nucleotide synthesized within cells using ATP, and it is under the action of a membrane-bound enzyme, adenylate cyclase. cAMP is continuously produced and inactivated by hydrolysis of 5′-AMP through a family of enzymes known as phosphodiesterase (12–14).

**Figure 2:**
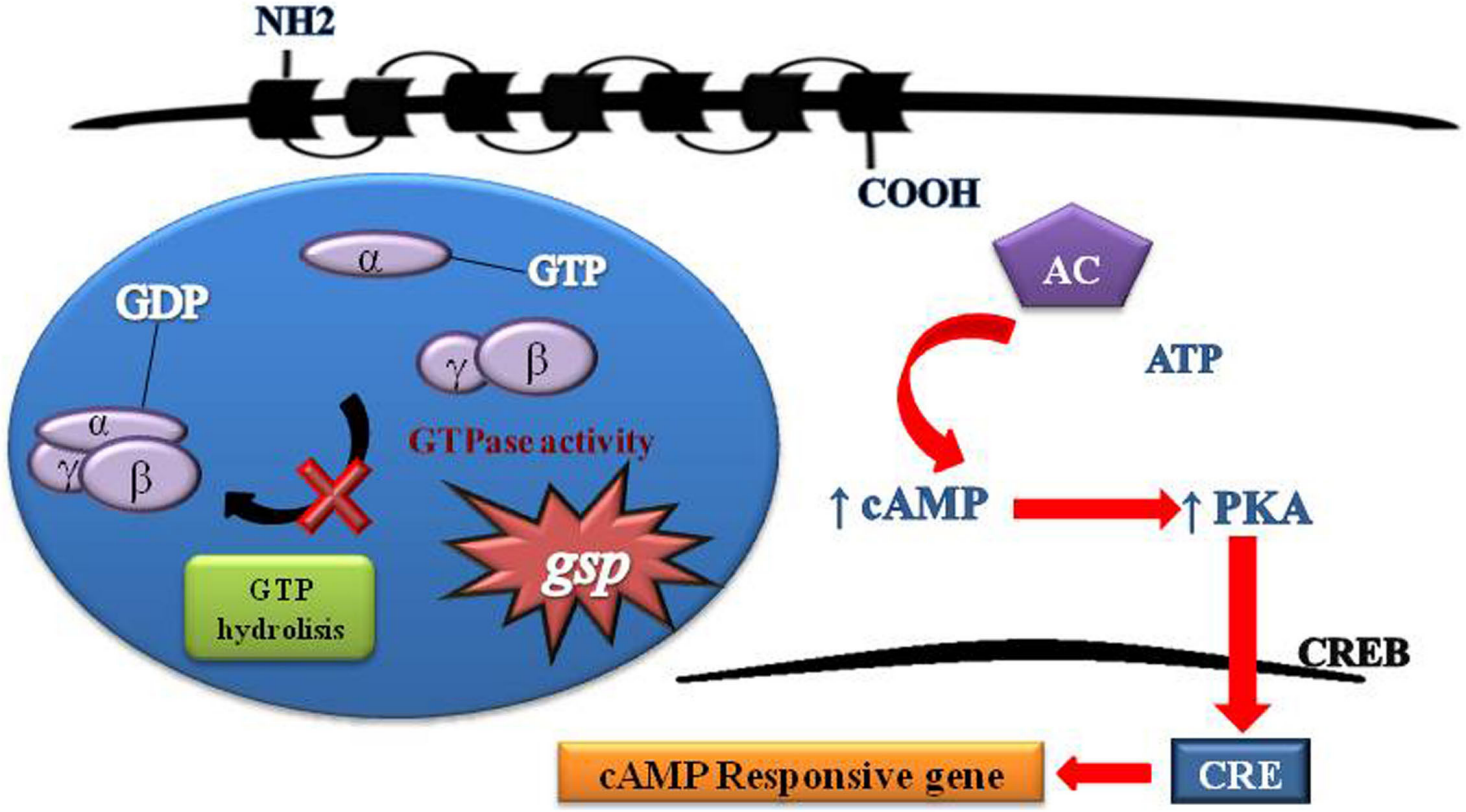
**Schematic representation of G protein activation and signaling**. Heterotrimeric G proteins are composed of three distinct subunits alpha, beta, and gamma. Activity G protein depends on the alpha subunit. The alpha subunit contains high-affinity binding sites for guanine nucleotides (GDP) and has intrinsic GTPase activity. The GDP-bound form binds tightly to beta and gamma units in its inactive state. The GTP-bound form dissociates from beta and gamma units and serves as a regulator of effectors proteins. The receptor molecules cause the activation of G proteins by facilitating the exchange of GTP for GDP on the alpha subunit. The duration of subunit separation is timed by the rate of alpha subunit-mediated hydrolysis of GTP. The *gsp* mutations Arg201Cys and Gln227Arg at exons 8 and 9, respectively, of GNAS causing a constitutive signal of cAMP pathway.

cAMP regulates many aspects of cell function, including enzymes involved in energy metabolism, cell division and differentiation, ion transport, ion channels, and contractile proteins. However, these effects are produced by a common mechanism, the activation of protein kinases by cAMP (Figure 2).

Proteins are phosphorylated by various protein kinases. The substrates of protein kinases and phosphatases include enzymes, neurotransmitter receptors, ion channels, and structural proteins, activating or inhibiting through phosphorylation, the target enzymes or ion channels. The increased cAMP production in response to the activation of β-adrenergic receptors affects several enzymes that are involved in glycogen metabolism, adipocytes, and muscle cells. The result consists of a coordinated response in which the energy stored as glycogen and fat becomes available as glucose, acting as a supply for muscle contraction (15).

cAMP, a second messenger, and its effector, protein kinase A (PKA), are key regulators of practically all cellular functions, such as cell growth and cell differentiation, and proliferation, and they mediate the effects of several hormones and neurotransmitters *via* GPCRs. This pathway is one of the major participants in the regulation of growth, proliferation, and hormonal secretion in adrenocortical cells (16, 17).

Once two cAMP molecules bind to each R subunit, the C subunits are released from the holoenzyme and can phosphorylate their targets, which are localized in the cytosol and in the nucleus. In adrenocortical cells, there is stimulation of glucocorticoid synthesis as well as transcriptional induction of steroidogenic enzymes and activation genes that are involved in cell replication (15, 18).

The majority of benign lesions of the adrenal cortex that lead to cortisol overproduction are linked to abnormalities of the cAMP pathway. Genetic defects in the cAMP/PKA that maintain pathway activation have been associated with adrenal disorders as follows:

(1) Ectopic expression of G protein-coupled receptors (19, 20).
(2) Somatic activating mutations in the gene coding for the Gα_s_ protein (*GNAS*) (6, 10, 21).
(3) Somatic activating mutations in the corticotropin receptor gene, *MC2R* (22, 23).
(4) Germline and/or somatic inactivating mutations in the gene coding for the RIα subunit of PKA (*PRKAR1A*) (13, 14, 18).
(5) Inactivating germline mutations in the genes coding two phosphodiesterases (PDE8 and PDE11A) (24, 25).
(6) Activating germline mutations in the gene coding for the Cα subunit of PKA (*PRKACA*) (26).
(7) Somatic mutations in PRKACA, which encodes the catalytic subunit of cyclic AMP-dependent protein kinase (27).
(8) Germline and somatic inactivating mutations of ARMC5 (28, 29).

Nevertheless, because these molecular events are not able to explain all cases of adrenocortical disorders, part of the molecular pathogenesis of the tumors causing Cushing’s syndrome remains a challenge to scientific researchers.

### *gsp* Mutations in Adrenal Cortical Tumors

Somatic mutations of genes encoding components of the cAMP/PKA pathway (*GNAS, PRKAR1A, PDE8B*) and β-catenin (*CTNNB1*) have been reported in a small subset of adrenocortical tumors that produce cortisol (27, 30).

The first report of the involvement of *gsp* mutations in adrenal tumors was by Lyons et al. In this study, the group tested the gene that encodes the alpha chain of Gi2, and the authors detected mutations that replaced arginine-179 with either cysteine or histidine in 3 of 11 tumors of the adrenal cortex and 3 of 10 endocrine tumors of the ovary (31).

Yoshimoto et al. systemically screened Gs alpha mutations in 197 human endocrine tumors. They included pituitary, thyroid, parathyroid, endocrine pancreas, and (cortex and medulla) adrenal tumors. They identified a unique, 29-year-old female patient with primary aldosteronism associated with a somatic *gsp* mutation in an aldosterone-producing adrenocortical adenoma. The authors commented that when the renin–aldosterone system is suppressed, aldosterone-producing adrenal adenomas become more sensitive to corticotropin stimulation. In this way, corticotropin is transmitted *via* Gs alpha-mediated cyclic AMP production. The study hypothesized that the *gsp* mutations may constitutively stimulate aldosterone synthesis in the glomerulosa zone, transmitting a constitutive signal *via* Gs-mediated cyclic AMP production, which would play an important role in the tumorigenesis of the aldosterone-secreting adenoma (3).

In 2000, Bugalho et al. described the presence of a mutation at codon 201 (CGT to TGT) in a patient with Cushing’s syndrome due to a functioning adrenal adenoma (32). In 2004, Dall’Asta et al. also identified *gsp* mutations in one patient with ACTH-independent Cushing’s syndrome. The presence of the *gsp* mutation seemed to alter the cortisol responses to agents *via* Gs protein-coupled receptors, whereas these responses are absent in other cases of adenoma-producing Cushing’s syndrome without *gsp* mutations (33).

Moreover, Libé and Bertherat investigated the presence of genetic alterations on a series of 10 ACTH-independent Cushing’s syndrome cases due to adrenocortical cortisol-secreting adenomas. The *gsp* mutation was identified in only one case, demonstrating that this abnormality is a rare cause of adrenocortical tumors. These findings suggest that different mechanisms are probably involved in adrenal tumorigenesis in primarily benign disorders (34).

A study conducted by Almeida et al. using the whole-genome expression profile (WGEP) of *PRKAR1A* and *GNAS*-mutant analysis revealed that not all cAMP activation is the same. Adrenal lesions harboring *PRKAR1A* or *GNAS* mutations share downstream activation of specific oncogenic signals (such as MAPK and cell cycle genes), but they differ substantially in their effects on others. These results support the hypothesis that several pathways can activate cAMP (13).

In 2013, Sidhu et al. described, for the first time, the presence of the p.R201C, a *GNAS* activating mutation in a malignant pediatric adrenocortical tumor. The malignant features described were as follows: areas of necrosis, microcytic degeneration, and both venous and capsular microinvasion. The tumor tissue also presented with abnormal allele-specific hypomethylation of the *KCNQ1OT1* gene involved in Beckwith–Wiedemann syndrome. Somatic mutations in these genes may constitutively activate the cAMP-protein kinase cascade, leading to cellular proliferation, which may then result in genomic instability and epigenetic alterations that give rise to ACTs and malignancy. This study suggested, for the first time, that activation of the cAMP-PKA cascade alone may not be sufficient to cause malignant transformation in the adrenal cortex without resulting in secondary events (35).

Recently, somatic mutations of *PRKACA* (encoding the catalytic subunit of PKA) have been identified in more than one-third of the patients with Cushing’s syndrome from sporadic adrenocortical adenomas; however, the molecular pathogenesis of the majority of sporadic adrenocortical tumors remains unclear (18, 27, 36).

In summary, apart from the known somatic mutations described in the literature, no other recurrent mutation by itself can explain the mechanism of tumor formation and hormone hypersecretion.

### *gsp* Mutations in Primary Macronodular Adrenal Hyperplasia

Over the last two decades, different studies have supported that multiple molecular mechanisms may be involved in the pathogenesis of PMAH (7, 12–14, 37). Several pathways were studied and analyzed for gene alterations, suggesting that there may be a heterogeneous group of diseases with a common presentation, ranging from subclinical hypercortisolism to overt Cushing’s syndrome.

Bilateral adrenal hyperplasia may be part of MAS that is associated with hypercortisolism, especially in young children during the first years of age. The adrenal nodules of these patients carried the *gsp* mutation and adrenal cortical cells increased the levels of cAMP (6, 8).

In 2003, Fragoso et al. identified two distinct *gsp* somatic mutations affecting arginine residues in codon 201 of *GNAS* in a few patients with PMAH without any features or manifestations of MAS (2).

Subsequently, Hsiao et al. also reported the presence of *gsp* somatic mutation in one additional patient with PMAH (4). On the other hand, an additional study failed to observe *gsp* mutations in PMAH (37). Technical variations in the methodologies employed for the investigation of *gsp* mutations could explain the discrepancy in these published findings.

Almeida et al. described the analyzed the WGEP of primary pigmented nodular adrenocortical disease (PPNAD) patients associated with gsp mutations. The data indicated that cAMP activation in adrenal lesions harboring *gsp* mutations share the downstream activation of some oncogenic signals but differ significantly in their effects on others (13).

Considering the great variation of MAS, these cases might characterize late *gsp* somatic mutations considering that similar mutations have been described outside the context of MAS (e.g., acromegaly, thyroid cancer, and ovarian–testicular neoplasms) (7).

Based on strong previous evidence implying that increased cAMP signaling is sufficient for cell proliferation and cortisol production, we posit that although these somatic activating mutations in *GNAS* are a rare molecular event, they are probably sufficient to induce the development of macronodule hyperplasia and variable cortisol secretion (12, 38, 39).

Primary macronodular adrenocortical hyperplasia is a heterogeneous disorder that could be associated with genetic defects in both germline and somatic levels. The presence of somatic *gsp* mutations was detected in rare cases with PMAH without MAS features. The role of *gsp* mutations in the development of this adrenal disorder remains partially unclear (2).

Recent studies have now indicated that PMAH is more frequently genetically determined than previously believed (28, 29). Germline mutations of *ARMC5* in ~50% of patients with apparently sporadic PMAH, and also in a large family with genetically transmitted PMAH. The *ARMC5* has no apparent link to the cAMP pathway, but its inactivation decreases the expression of both MC2R and various steroidogenic enzymes (19).

### Activation of Cyclic AMP Signaling in Lesions of the Adrenal Cortex due to Somatic GNAS Mutations

Aberrations in cAMP/PKA signaling are essential to the pathogenesis of benign cortisol-producing lesions of the adrenal cortex (40).

An important study by Almeida and coauthors analyzed the WGEP of adrenal lesions harboring somatic *GNAS* mutations in the normal adrenal pool and tissue with somatic *PRKAR1A* genes. They included three microdissected samples from adrenal lesions that were all caused by the same somatic *GNAS* mutation (p.R201H) as follows: a cortisol-producing adenoma from patients with PMAH and Cushing’s syndrome and hyperplasia from patients with MAS and Cushing’s syndrome. The results of this study showed that the MAPK and p53 signaling pathways were highly overexpressed in all lesions compared with normal tissues. *GNAS*-mutant tissues were significantly enriched for extracellular matrix receptor interaction and focal adhesion pathways compared with *PRKAR1A* mutants. In addition, *NFKB, NFKBIA*, and *TNFRSF1A* were overexpressed in *GNAS*-mutant adrenal tissue (13).

*NFKB* is a transcription activator nuclear factor kappa light-chain enhancer of activated B cells, a complex protein that controls DNA transcription, cytokine production, and cell survival. The nuclear factor kappa–beta controls the expression of various genes that are involved in cancer-related processes, including immune and inflammatory responses; cell adhesion, proliferation, differentiation, and apoptosis; and angiogenesis (13).

*TNFRSF1A* is a tumor necrosis factor receptor member of the superfamily 1A and the protein encoded by this gene is a member of the TNF-receptor superfamily. This protein is one of the major receptors for tumor necrosis factor-alpha. This receptor can activate NF-kappa–beta and mediate apoptosis, and it functions as a regulator of inflammation (13).

The study of adrenal lesions harboring *PRKAR1A* or *GNAS* mutations, conducted by Almeida et al. suggested that cAMP signaling inhibitors could be used as molecularly designed therapies for subclinical CS in the context of bilateral adrenal hyperplasia (13).

Despite the importance of the cAMP pathway, these molecular events are a rare cause of adrenocortical tumors and adrenal hyperplasia, showing that several other genes should be involved in the cAMP pathway as long as other genes are involved in adrenal tumorigenesis and hyperplastic development.

## New Insights

This review aimed to provide a summary of the main studies on the role of cAMP and *gsp* mutations on the development of adrenocortical tumors and adrenal hyperplasia. Throughout this review, we showed that *gsp* mutations are a rare cause of adrenal disorders and involvement of *gsp* mutations in increasing the cAMP levels is not always detected.

The molecular pathogenesis of cortisol-producing adrenal adenomas and adrenal hyperplasia is not well understood.

Somatic mutations in the gene encoding beta-catenin (*CTNNB1*) have primarily been found in non-secreting adrenocortical adenomas, and there is some evidence that increased endocrine activity may be linked to aberrant cAMP signaling.

Tumor suppressor genes point mutations (such as *CTNNB1*) and alterations in the cortisol producing (*ARMC5* and *PKC* mutations) could lead to alterations in cAMP pathway.

Mutations in the genes *PDE11A, PDE8B*, and *PRKAR1A* have also been identified in patients with adrenal disorders related to cortisol production (14).

Nevertheless, lesions of the adrenal gland that are associated with adrenal Cushing syndrome, independent of their *GNAS, PRKAR1A, PDE11A*, and *PDE8B* mutations, have functional abnormalities in cAMP signaling. It is hypothesized that epigenetic events or additional genetic defects of the regulatory molecules in this pathway exist and have yet to be identified (41).

## Conclusion

The genetic analyses of the molecular pathogenesis of sporadic cortisol-secreting adrenocortical adenomas confirm the key role of the cAMP/PKA-signaling pathway in stimulating both the function and proliferation of adrenocortical cells. They provide insights into the development of adrenal hormonal autonomy and may provide the basis for novel advances in the diagnosis and therapy of adrenal Cushing’s syndrome.

In conclusion, this review demonstrates that not all increased cAMP/PKA signaling has the same effect on adrenocortical tumor formation. The role of non-PKA-dependent functions of cAMP in the adrenal cortex has not been adequately investigated.

Based on strong previous evidence implying that increased cAMP signaling is sufficient for cell proliferation and cortisol production (38), we surmise that although really rare, these *gsp* mutations are probably sufficient for macronodule formation and the hypercortisolism status.

## Author Contributions

MCBVF, IQW, IPC, BMPM: All authors contributed equally for the article.

## Conflict of Interest Statement

The authors declare that the research was conducted in the absence of any commercial or financial relationships that could be construed as a potential conflict of interest.
